# Falsification of home rapid antigen lateral flow tests during the COVID-19 pandemic

**DOI:** 10.1038/s41598-024-53383-8

**Published:** 2024-02-09

**Authors:** Devashish Ray, Raenhha Dhami, Jan Lecouturier, Laura J. McGowan, Aritra Mukherjee, Ivo Vlaev, Michael P. Kelly, Falko F. Sniehotta

**Affiliations:** 1 NIHR Policy Research Unit in Behavioural Science-Population Health Sciences Institute, Faculty of Medical Sciences, Newcastle University, Newcastle Upon Tyne, UK; 2 NIHR Policy Research Unit in Behavioural Science-Behavioural Science Group, Warwick Business School, University of Warwick, Coventry, UK; 3 Primary Care Unit, Department of Public Health and Primary Care, University of Cambridge, Forvie Site, Cambridge, CB2 0SR UK; 4 Department of Public Health, Preventive and Social Medicine, Center for Preventive Medicine and Digital Health Baden-Wuerttemberg, Heidelberg University, Heidelberg, Germany; 5 Biostatistics Research Group, Population Health Sciences Institute, Newcastle University, Newcastle Upon Tyne, UK

**Keywords:** Psychology, Diseases

## Abstract

During the COVID-19 pandemic, lateral flow tests (LFTs) were used to regulate access to work, education, social activities, and travel. However, falsification of home LFT results was a concern. Falsification of test results during an ongoing pandemic is a sensitive issue. Consequently, respondents may not answer truthfully to questions about LFT falsification behaviours (FBs) when asked directly. Indirect questioning techniques such as the Extended Crosswise model (ECWM) can provide more reliable prevalence estimates of sensitive behaviors than direct questioning. Here we report the prevalence of LFT FBs in a representative sample in England (n = 1577) using direct questioning (DQ) and the ECWM. We examine the role of demographic and psychological variables as predictors of LFT FBs. We show that the prevalence estimates of the FBs in the DQ condition were significantly lower than the ECWM estimates, e.g., reporting a negative result without conducting a test: 5.7% DQ vs 18.4% ECWM. Moral norms, subjective norms, anticipated regret, perception of risk to self, and trust in government predicted some of the FBs. Indirect questioning techniques can help provide more realistic and higher quality data about compliance with behavioural regulations to government and public health agencies.

## Introduction

Large scale home testing using rapid antigen lateral flow tests (LFTs) was one strategy used to contain the spread of the COVID-19 virus in England, and globally^1^. Between April 2021 and March 2022, everyone in England was provided access to free LFTs. The government urged the public to use LFTs for twice-a-week testing, even when asymptomatic, and to report results of all tests (irrespective of the result) within 24 h either online through a government website or through a 24-h phone helpline^2^. Those who tested positive were required to self-isolate immediately. Proof of a recent (taken within 48 h) negative result was introduced as a requirement to gain access to work and educational settings, to indoor and outdoor mass events, and for travel^3^. Regular COVID-19 testing was framed as a civic and moral duty to family, friends, and society; however, compliance with testing and managing the consequences of a positive or negative result sometimes generated competing social obligations and ethical dilemmas^4^.

As the use of LFTs became widespread practice, there were media reports of some falsification of LFT results, e.g. to gain entry to events^5^, to avoid travel restrictions^6^, or to avoid school or work^7^. Analyses of these reports identified four distinct types of falsification behaviours (FBs): reporting a negative test without doing a test (*FB*1); reporting a positive test result as negative (*FB*2); reporting a positive test after having produced a fake positive test (*FB*3); and sharing information about the test for someone else to report it as their own test (*FB*4). The possibility that falsification of self-test results could undermine efforts to control the pandemic was a potential public health problem^8^. Success of a mass testing programme requires individual participation and commitment. Hence understanding the factors that can explain behavioural variation through applied behavioural science is potentially important to help with efforts to increase compliance. For these purposes, it is necessary to have *reliable* estimates about the extent of LFT falsification behaviours (FBs) and identify factors that may account for individual differences in non-compliance with guidance on testing and reporting of LFT results.

Previous studies have highlighted that individuals who perceive a high risk of contracting or being harmed by the COVID-19 virus, seek out more information, and feel a moral obligation to comply are more likely to report compliance with transmission reducing behaviours (TRBs)^9–13^. Compliance with COVID-19 TRBs has been reported to be higher also in certain sociodemographic characteristics, such as older people, women, and persons who have attended higher education^14–16^. Men and younger individuals may be more prone to non-compliance^9,17–21^. Individuals may also lack the practical capacity to comply due to their occupation (e.g., working in a key sector) or economic constraints^20,22,23^.

Behavioural theories can help to understand and predict LFT FBs. Two theories that have been widely used to investigate (non)compliance with COVID-19 TRBs are the protection motivation theory^24^ and an extended theory of planned behaviour (TPB)^10,25^. The PMT has been demonstrated to predict engagement with UK government’s guidance on COVID-19 testing using LFTs^21,26^, as well as adherence to various COVID-19 TRBs (e.g., hand washing, wearing face covering, and physical distancing) during the pandemic^27,28^. Other studies have demonstrated the utility of an extended TPB (by integrating additional theoretical constructs) to predict non-compliance with COVID-19 TRBs^10,12,29^; these additional constructs include moral norms (personal internalised moral rules)^30^, descriptive norms (individual’s beliefs about what other people do)^25^ and anticipated regret (negative emotions that regret brings forth when individuals act or fail to act in a certain way and begin to think counterfactually)^31^. Motivation to comply with COVID-19 protective behaviours may also be influenced by level of trust and confidence in government institutions to tackle the pandemic^32,33^.

**Figure 1:**
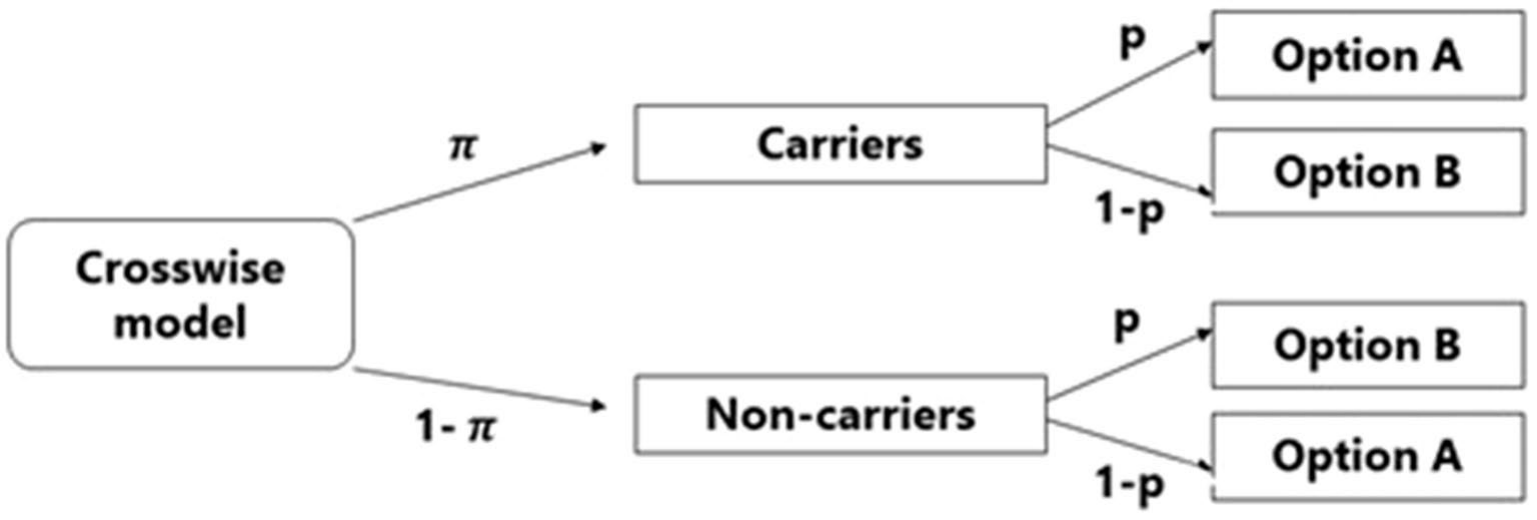
Tree diagram of the Crosswise model (Yu et al.^36^); π represents the unknown prevalence of the behaviour of interest; p denotes the known prevalence of the non-sensitive question (the randomisation probability); option A = Yes (or No) to both questions; option B = Yes to one and No to the other, irrespective of which one.

Falsification of diagnostic test results during an ongoing pandemic has moral, social and medical implications^34^. Consequently, respondents might not answer truthfully to direct questions (DQ) about FBs, even when assured of anonymity, due to social desirability bias^35^. To elicit more honest responding, indirect survey questioning techniques such as the crosswise model (CWM) provide an alternative to DQ, controlling for social desirability bias^36^. The CWM (represented in Fig. 1) maximises the confidentiality of individual answers by adding a randomisation procedure to the questioning. It presents respondents with the sensitive question (e.g., have you ever falsified a COVID-19 LFT) alongside an unrelated non-sensitive question (e.g., is your mother’s birthday in May, June, or July?), the prevalence of which (the “randomisation probability”) can be known from official birth statistics. Respondents are instructed to indicate whether “my answer is Yes (or No) to both questions (option A), or “my answer is Yes to one and No to the other, irrespective of which one” (option B). Neither of these response options reveals whether the respondent carries the sensitive attribute. This is expected to increase the respondent’s motivation to answer truthfully. However, since the probability of selecting option A can be obtained, the prevalence of the sensitive behaviour can be estimated, but only at group level. Importantly, the CWM does not provide any “safe” self-protective response option that respondents could choose to explicitly deny having engaged in the behaviour. However, like any questioning technique, the validity of the CWM rests on the assumption that participants adhere to the model’s instructions. The CWM does not provide a method to detect instruction non-adherence. In this study, we used the extended CWM (ECWM)^37^ a recent advancement of the CWM that has been favourably evaluated^38^. In the ECWM, the standard CWM is extended by randomly assigning participants to one of two non-overlapping groups. In both groups, respondents receive the same sensitive question but a different non-sensitive question. The advantage of the ECWM is that in addition to assuring anonymity of responses, it allows for the detection of instruction non-adherence, as explained in “Methods” section.

**Figure 2:**
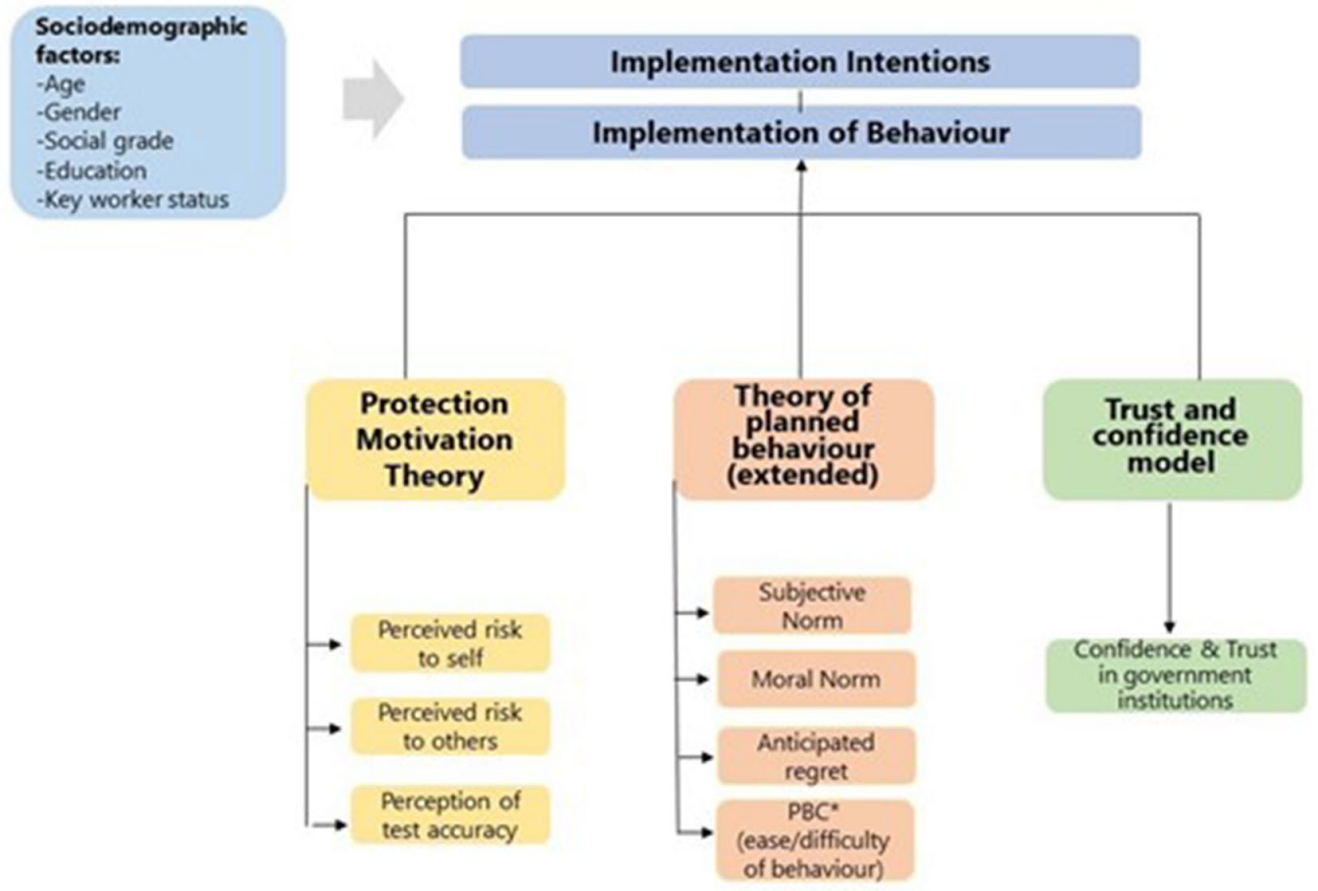
A diagram showing the theoretical models that were used in this study to help explain and predict COVID-19 lateral flow test falsification behaviours. The predictor variables that were investigated are displayed. [*PBC perceived behavioural control].

Based on this introduction, the current study had two objectives. The main objective was to investigate the proportion of people in England that engaged in home COVID-19 LFT FBs at least on one occasion. The secondary objective was to identify psychological and demographic correlates of FBs. We predicted that ECWM would lead to higher estimates for the FBs compared to DQ. We hypothesised that the following psychological constructs would have a negative association with FBs: perceived risk (to self and to others) from COVID-19 infection, beliefs about accuracy of LFTs, subjective norms, moral norms, perceived behavioural control (ease/difficulty of performing the behaviour), and anticipated regret. With respect to demographic variables, we predicted that younger people and men would be more prone to engaging with LFT FBs. Figure 2 shows the psychological and demographic variables that were probed for their potential role as predictors of the FBs.

## Results

### Prevalence estimates of the falsification behaviours

When asked directly, 5.7% of the respondents indicated they had reported a negative result without doing a test (*FB*1) and 4.5% indicated they reported a positive test result as negative (*FB*2). Only 1.7% declared that they reported a positive result after having produced a fake positive test (*FB*3), and only 2% declared that they shared information about their test with someone else (*FB*4). The findings are shown in Table 1.

**Table 1:** Prevalence for the four test falsification behaviours (FBs) obtained through direct questioning (DQ).

Description of the behaviour	Prevalence (in %)
FB1: reported a negative test result without doing a test	5.736
FB2: reported a positive test result as negative	4.590
FB3: reported a positive test after having produced a fake positive test	1.714
FB4: shared information about the test for someone else to report it as their own test	2.099

The prevalence (in %) was computed by dividing the total number of “Yes” responses by the total number of overall responses for each FB in the DQ condition.

In the ECWM condition, the likelihood ratio tests (performed to compare the prevalence estimates between the two ECWM groups) for *FB1*, *FB2*, and *FB3* were not statistically significant, indicating instruction adherence and reliability of the estimates. Therefore, the pooled ECWM prevalence estimates for *FB*1, *FB*2, and *FB*3 are considered valid. For *FB*4, the likelihood ratio test was statistically significant (P < 0.001) indicating significant difference in the estimates between ECWM1 and ECWM2. The analyses revealed instruction non-adherence presenting as systematic preference for option B by ECWM1 participants. Hence, the ECWM prevalence estimates of *FB*4 were considered unreliable. These findings are shown in Table 2.

**Table 2:** Prevalence estimates for the four test falsification behaviours (FBs) obtained using the Extended Crosswise model (ECWM).

Description of the behaviour	Groups	Estimate of $\pi$	Std. error	Likelihood-ratio test		
				*G2*	*df*	*p *value
FB1: Reported a negative test result without doing a test	ECWM 1	0.214	0.044	0.946	1	0.331
	ECWM 2	0.154	0.043			
	**Pooled estimate**	**0.184**	0.031			
FB2: Reported a positive test result as negative	ECWM 1	0.086	0.043	0.592	1	0.442
	ECWM 2	0.132	0.043			
	**Pooled estimate**	**0.110**	0.030			
FB3: Reported a positive test result after having produced a fake positive test	ECWM 1	0.060	0.042	0.022	1	0.882
	ECWM 2	0.068	0.042			
	**Pooled estimate**	**0.064**	0.030			
FB4: Shared information about the test for someone else to report it as their own test	ECWM 1	0.320	0.046	20.801	1	0.000 (P < 0.001)
	ECWM 2	0.038	0.041			
	Pooled estimate	0.178	0.031			

$\pi$ represents the prevalence estimate of the FB for the two ECWM groups, ECWM1 and ECWM 2. Likelihood-ratio test was performed to compare the prevalence estimates between the two ECWM groups, to detect instruction non-adherence amongst participants.

Significant values are in [bold].

**Figure 3:**
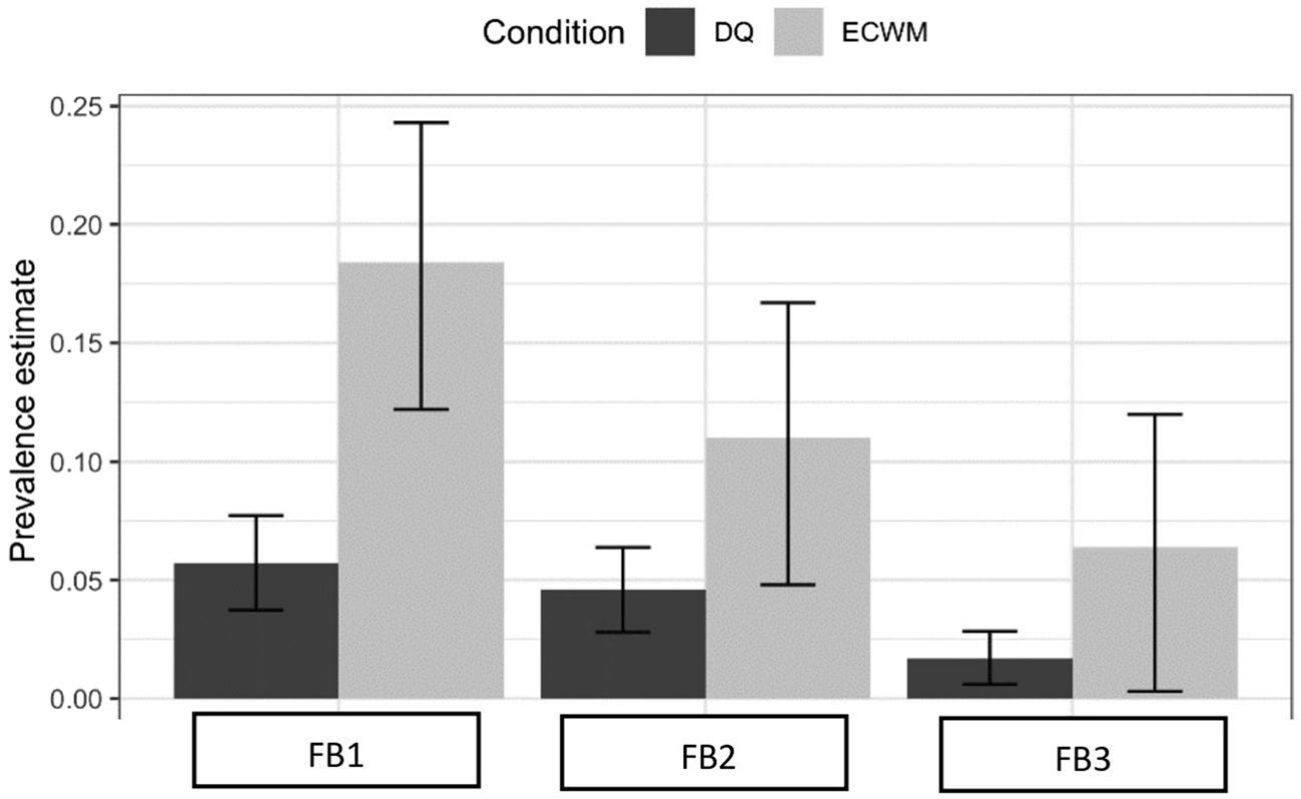
Box plot showing prevalence estimates of falsification behaviours (*FB*): *FB*1, *FB*2, and *FB*3. The black bars represent prevalence data obtained from direct questioning (DQ) and the grey bars represent prevalence estimates obtained using the Extended Crosswise model (ECWM). The error bars represent 95% confidence intervals.

As shown in Table 3 and Fig. 3, when the questions were asked indirectly (the ECWM group), prevalence estimates were significantly higher (p < 0.001) than those obtained through DQ for the following three behaviours: *FB*1 (18.4% ECWM vs 5.7% DQ), *FB*2 (11% ECWM vs 4.6% DQ), and *FB*3 (6.4% ECWM vs 1.7% DQ).

**Table 3:** Comparison of the prevalence estimates obtained through direct questioning (DQ) and the extended crosswise model (ECWM) by using the two-proportion Z test.

Behaviour	Prevalence estimates (%)		Two-proportion Z test		
	DQ	ECWM	*Test-statistic*	*df*	*p *value
*FB*1: Reported a negative test result without doing a test	5.73	18.4	43.59	1	< 0.001
*FB*2: Reported a positive test result as negative	4.59	11.0	15.526	1	< 0.001
*FB*3: Reported a positive test result after having produced a fake positive test	1.71	6.4	14.553	1	< 0.001

Respondents identified plausible reasons a person may be motivated to engage in FBs. Around 75% of the respondents identified “not wanting to miss a holiday that was already booked and paid for” and “not wanting to self-isolate”, as potential motivators for reporting a positive test result as negative. A large proportion of respondents (84%) identified “wanting to stay off work” as a reason for reporting a positive test result after generating a fake positive test (see Supplementary Figs. S1 and S2 online). Figure S1 shows participants’ perceptions of factors that may motivate people to report a positive test result as negative, as a bar graph. Figure S2 shows participants’ perceptions of factors that may motivate people to report a negative test result as positive, as a bar graph.

### Association between falsification behaviours and demographic variables

After excluding incomplete data, analysis was conducted on 1405 respondents. Age was the only statistically significant association for demographic variables, which showed that the odds of “reporting a positive test result after producing a fake positive test” (*FB*3) *decreases* as age *increases* (OR 0.176 [95% CI 0.034, 0.903], *p* = 0.023). The findings pertaining to other demographic variables (gender, social grade, key worker status, and education level) are shown in Supplementary Table S2 online.

### Association between falsification behaviours and psychological variables

The analyses showing the associations between the FBs and psychological variables are presented in Table 4. Subjective norms (OR 0.69, 95% CI [0.49,0.98]) and moral norms (OR 0.55, 95% CI [0.38, 0.79]) were statistically significant for *FB*1 and *FB*2, respectively. For *FB3*, perception of risk (to self), confidence in government institutions, subjective norms, moral norms, and anticipated regret were statistically significant. Specifically, there was a *negative* association between engaging in FBs and moral and subjective norms (odds-ratio < 1), implying that those who perceived the behaviours as morally wrong and believed that people close to them would disapprove, were less likely to engage in FBs. We noted that for *FB*3, perception of risk (to self) from COVID-19 infection was associated with *increased* likelihood of producing a fake positive test (OR 2.34, 95% CI [1.12, 4.91]). It may be that those who perceived themselves to be at high risk of catching COVID-19 (e.g., at their work setting) engaged in this *FB* as an act of self-protection; reporting a (false) positive test would warrant self-isolation during the ongoing pandemic. This idea is supported by the finding that 84% of our survey respondents believed that people would falsely declare positive COVID-19 status to secure time off work.

**Table 4:** Association between the falsification behaviours and psychological variables.

Psychological variables	Odds ratio	95% CI		p value
		Lower	Upper	
*FB*1: Reported a negative result without doing a test				
Perception of risk (self)	1.167	0.824	1.655	0.379
Perception of risk (others)	1.143	0.831	1.572	0.409
Confidence in govt	0.88	0.633	1.222	0.442
Belief in test accuracy	0.978	0.693	1.379	0.898
Perceived ease of falsifying	1.161	0.836	1.614	0.376
**Subjective norm**	**0.699**	**0.496**	**0.986**	0.038*
Anticipated regret	0.764	0.515	1.135	0.185
Moral norm	0.674	0.462	0.983	0.053
*FB*2: Reported a positive test result as negative				
Perception of risk (self)	1.459	0.98	2.174	0.062
Perception of risk (others)	1.028	0.698	1.512	0.89
Confidence in govt	0.896	0.596	1.347	0.595
Belief in test accuracy	1.288	0.881	1.884	0.211
Perceived ease of falsifying	1.117	0.736	1.694	0.609
Subjective norm	0.632	0.408	0.978	0.055
Anticipated regret	0.642	0.408	1.01	0.091
**Moral norm**	**0.551**	**0.382**	**0.796**	0.004**
*FB*3: Reported a positive result after having produced a fake positive test				
**Perception of risk (self)**	**2.349**	**1.123**	**4.912**	0.027*
Perception of risk (others)	1.508	0.71	3.201	0.299
**Confidence in govt**	**0.52**	**0.284**	**0.954**	0.047*
Belief in test accuracy	1.396	0.56	3.48	0.43
Perceived ease of falsifying	0.544	0.278	1.064	0.072
**Subjective norm**	**0.356**	**0.201**	**0.628**	0.000***
**Anticipated regret**	**0.381**	**0.222**	**0.654**	0.002**
**Moral norm**	**0.379**	**0.224**	**0.640**	0.001**

Modified logistic regression from RRreg package across the three sensitive behaviours was performed while controlling for questioning format (added as an effect-coded variable). This modified regression approach accounts for the discrepancy between the observed responses and the true latent states in the E(CWM).

*p < 0.05; **p < 0.01; ***p < 0.001.

We found that people were *less* likely to engage in *FB3* (false declaration of COVID-positive status) when levels of confidence in government institutions (OR 0.52, 95% CI [0.28, 0.95]) and anticipated regret (OR 0.38, 95% CI [0.22, 0.65] were *high* (odds-ratio of < 1 for both). However, these two factors did not have statistically significant associations with *FB*1 and *FB*2 (false declaration of COVID-negative status). The underlying reason(s) for these observations is not clear. It is possible that this pattern of associations may be related to the nature of these behaviours. One could argue that *FB3* has a more active component (adding a liquid to the test strip to generate a fake positive test), whereas *FB1* and *FB2* are relatively passive. No statistically significant associations were found between the *FBs* and perception of risk to others, beliefs about usefulness and accuracy of LFTs, and perceived ease of falsification of self-reported test results.

We also tested if the effects of the predictors were moderated by the questioning format by incorporating the latter as an effect-coded interaction. The analyses showed that the relationship between subjective norm and *FB*1 was affected by social desirability bias in the DQ format. The results are shown in Supplementary Table S3 online.

## Discussion

This study addresses an important research and evidence gap. Based on a large representative sample of the adult population in England, this study is the first to present evidence on the nature and extent of falsification of home COVID-19 LFTs. Home testing using LFTs for rapid diagnostic and screening purposes was an important public health strategy to protect the vulnerable and control the spread of infection during the pandemic^39^. It is therefore problematic that a substantial proportion of the population engaged in LFT FBs. Prevalence estimates using the ECWM, which provided increased anonymity, were significantly higher than those derived from DQ; e.g., the ECWM estimate for FB1 (reporting a negative result without doing a test) was higher than the DQ estimate by 12 percentage points. The results indicate that COVID-19 LFT falsification was perceived as a sensitive topic, and participants’ responses to direct questions were affected by social desirability. Further, the findings suggest that it is likely that the ECWM successfully controlled the influence of social desirability and presented a more realistic and valid insight into peoples’ FBs. A study that compared DQ and ECWM to estimate prevalence of compliance with COVID-19 guidance on handwashing reported a similar pattern of results^40^.

False declaration of COVID-negative status (*FB*1 and *FB*2) is important because of the potential adverse effects for one’s health, and for the health of others. It is likely that a proportion of people who reported a negative result without doing a test were asymptomatic carriers and a source of transmission of the virus^41^. Sharing screenshots of a negative LFT test with others (*FB*4) may have played a role in many people who attended a recreational event in Netherlands to test positive for COVID-19 despite entry to the event requiring proof of a recent negative LFT result or a COVID-19 vaccination certificate, according to media reports^5^. Our results show that false declaration of COVID-positive status (*FB*3) was less prevalent than false declaration of COVID-negative status (*FB*1 and *FB*2). Individuals who engaged in *FB*3 were more likely to be of younger age, and presumably did so for the purpose of taking time off work. Although *FB*3 was less prevalent, we know from media reports that engaging in false declaration of COVID-positive status could have harmful consequences for the individual (e.g. loss of job and criminal penalty), and for the employer organisation (e.g. economic losses)^42,43^, so it is an important issue to address.

The symmetric answering structure of the ECWM is expected to have encouraged honest responding (as no opportunity to resort to a “safe” answering option is available), thereby strengthening the validity of the prevalence estimates^37^. Further, the ECWM enabled us to detect instruction non-adherence and indicate whether the obtained prevalence estimates are trustworthy^37^. It is not possible to know the exact reason for the instruction non-adherence or the extent of it. One factor that may have contributed to this in our study is the change in the attribute of the non-sensitive question for *FB*4 (switching from asking for the birth month of a close family member to that of a person from a wider group—sibling, friend, or a person they know—the choice of which was left at the discretion of the respondents). This may have led to misunderstanding by participants, leading to instruction non-adherence. However, the ECWM can only detect instruction non-adherence that occurs due to a systematic preference for one of the two response options. Like other questioning techniques, the ECWM cannot detect non-adherence that may occur if respondents do not sufficiently understand the instructions or simply do not pay attention^44^.

Despite the advantages of the ECWM, there are some methodological challenges of the model that researchers should be aware of. First, an obvious limitation of the EWCM (and other indirect questioning techniques) is that prevalence of behaviours can only be analysed at group level and not at individual level. Second, random error introduced by the randomisation question in the (E)CWM results in a greater sampling variance and lower statistical power than conventional DQ method. Hence, to obtain confidence intervals commensurate with DQ, the (E)CWM requires larger sample size to increase power^37^. Third, methods to compute correlation and regressions involving the (E)CWM are statistically complex and require special software^45^. Finally, a prerequisite for obtaining valid results is that respondents fully understand the procedure and comprehend the questions^44^. Therefore, careful procedural implementation of the ECWM (namely, the wording and choice of the randomisation questions and instructions) is essential.

The study findings provide an insight into the various factors to take into consideration while framing directives, to increase cooperation and compliance. Our results show that subjective and moral norms, and anticipated regret predicted disengagement from some of the FBs. The importance of these predictors in explaining individual-level variation in behaviours is highlighted in research on (non)compliance with different COVID-19 protective behavioural measures^13,28,46^. The effects of subjective and moral norms suggest that messages promoting moral obligation and social responsibility for preventing transmission of the virus to vulnerable others (e.g., by self-isolating if tested positive) may facilitate greater intention to follow guidance for testing and recommended post-test behaviours^22^. Heightening the level of anticipated regret using messages that have both cognitive (e.g. information about risks) and emotional appeal (e.g. threat, fear) may strengthen the intention to honestly disclose self-test results^47^. The role of trust in government institutions as a predictor of intention to adhere to COVID-19 behavioural measures is shown in several UK studies^21,32,48^. This finding implies the need for clear information and unambiguous health instructions that expresses government transparency and effective communication^33^.

In this study, perceived *public* threat of COVID-19 was not strongly associated with any of the FBs. Instead, perceived *personal* threat was shown to predict “reporting a positive result after producing a fake positive test”, suggesting that self-interest was a strong motivator for engaging with this particular behaviour. This finding is in contrast to evidence from several studies which suggest that—in addition to protecting the self—prosocial motivations (a desire to promote the welfare of others and adhere to social norms) are strong facilitators of compliance with recommendations during an ongoing pandemic^9,32,49,50^. The literature suggests that both self-interest and prosocial framing of messages can equally promote intention to comply with COVID-19 protective behaviours and regulations^51,52^. Taken together, these findings suggest that persuasive health communications aimed at increasing compliance with health protection behavioural measures are likely to be effective if they emphasise benefits to self in addition to benefits to others.

For researchers, an indirect questioning technique such as the ECWM offers a promising method for estimating prevalence of sensitive behaviours and attitudes where estimates derived from DQ are likely to be distorted by social desirability bias^53^. It is important that public health practitioners and policy decision-makers are aware of the existence and consequences of such a bias. Data about compliance with behavioural regulations derived from conventional DQ methods can considerably affect the quality of the evidence available to policymakers and public health experts in their decision making^54^. Indirect questioning techniques can help provide more realistic and higher quality data to government and public health agencies.

The study findings also emphasise the importance of bioengineering efforts that are underway to create the next generation of high accuracy and easy-to-use LFTs and digital readers with remote diagnostic capability, which will eliminate the need for self-reporting of test results. An ‘artificial-intelligence powered’ LFT reader that is currently undergoing pilot-testing in the UK is capable of—in combination with the users’ smartphone camera and a phone-based app—data capturing and providing digitally connected and auditable LFT results^55^. These technological innovations are significant because LFTs can play a vital role in decentralised and affordable self-testing not only during a viral pandemic but also in the detection of other infectious diseases of epidemic potential^56^.

## Methods

### Study design

Ethical approval for this study was obtained from Newcastle University Research, Policy, Intelligence and Ethics Team on 8 July 2022 with reference 24446/2022. We have complied with all relevant ethical regulations. Online informed consent was obtained from all participants included in the study. Participants were compensated for their participation by the panel provider.

### Study participants

Eligible participants were adults (18 years and older) living in England who had previously taken a home LFT for COVID-19. Recruitment of participants and online delivery of the survey (between 23rd and 30th September 2022) was managed by YouGov^®^ market research company. YouGov^®^ used quota sampling to generate a sample that was representative of the population that met the eligibility criteria and ensure that respondents in the two ECWM groups were matched by age, gender, and other demographic factors. Comparison between the two questioning groups (DQ and ECWM) also did not indicate significant differences with regard to age group or gender. The details of the characteristics of survey respondents can be found in Supplementary Table S1 online.

### Sample size

There was no formal a priori power analysis conducted, however, we aimed to collect N > 1200 for 80% power at 0.05 level of significance based on Ulrich et al. power calculations for comparing the two questioning formats and the calculation of the prevalence estimates^57^. Participants (n = 1577) were randomly assigned to one of three groups: DQ (n = 530) and two ECWM groups (ECWM1, n = 523 and ECWM2, n = 524). Almost twice as many respondents were allocated to the ECWM condition (1047) than to the DQ condition. This was to compensate for the lower statistical power of the EWCM as a result of the randomisation procedure which introduces additional variance to the estimates, thereby inflating their standard error^57^.

### Survey design

The four behaviours of interest were formulated as sensitive questions as follows—*FB*1: have you ever reported a negative result without doing a test?; *FB*2: have you ever reported a positive result as negative?; *FB*3: have you ever reported a positive result after having produced a fake positive test (e.g., by using liquids such as a soft drink or other drink)?; and *FB*4: have you ever shared information of your LFT in order for someone else to report it as their own test (e.g., giving them the test strip ID or a picture of the test strip)?

**Figure 4:**
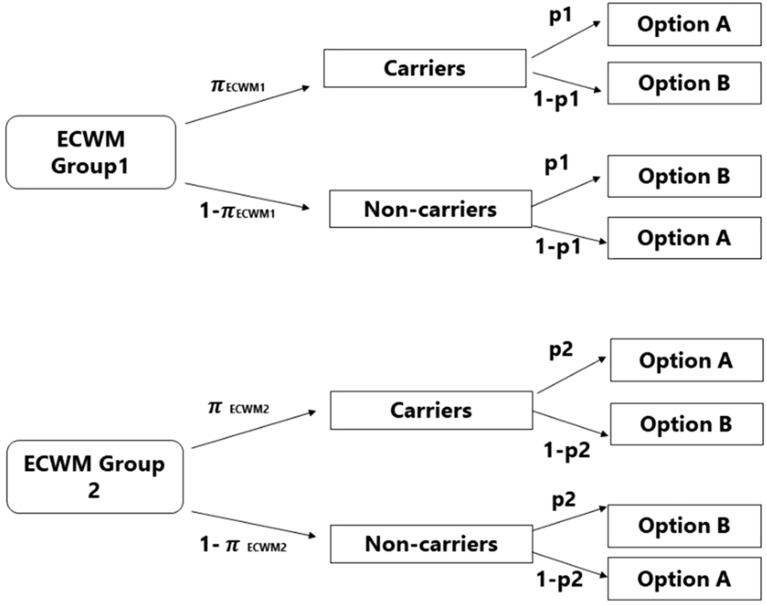
Tree diagram of the Extended Crosswise model (ECWM) (Heck et al.^37^). π represents the unknown prevalence of the sensitive behaviour; the two ECWM groups receive a different non-sensitive question of known prevalence: p for one group and complementary prevalence p2 (p2 = 1 − p) for group 2. Hence, the response alternatives shown on the right side of the model are swapped in ECWM group 2 compared to ECWM group 1.

DQ participants were directly asked the sensitive questions (e.g., have you ever reported a negative LFT result without doing a test?) and had to respond with either “*Yes*” or “*No*”. As shown in Fig. 4, for ECWM participants, each sensitive question was paired with a non-sensitive question (the randomisation question). The non-sensitive question differed between the two groups, so that the randomisation probability used in ECWM1 (e.g., is your mother’s birthday between May and July?) was complementary to the probability used in ECWM2 (e.g., is your mother’s birthday between August and April?). ECWM participants responded to the sensitive question and the randomisation question simultaneously, by indicating that their answer is either “my answer is Yes (or No) to both questions” (option A), or “my answer is Yes to one and No to the other, irrespective of which one” (option B). We used different randomisation questions for each sensitive question due to the possibility that some respondents may get suspicious if the randomisation items are iterations of the same (i.e., mother’s birth month)^58^. However, the months used for all the randomisation questions were kept the same, to minimise the likelihood of instruction non-adherence^37^. The implementation of the ECWM in this study is shown in Supplementary Methods. The survey questionnaire items were informed by theory and existing research on non-compliance with COVID-19 protective behaviours. Multi-item self-report measures of constructs based on measures and scales used in previous studies^10–12,21,28,29^ were used to collect data on psychological predictor variables. The full survey questionnaire is available at https://osf.io/2kcuw/.

### Statistical analysis

Data and analysis scripts are available at the Open Science Framework (OSF; https://osf.io/2kcuw/). Observed answer frequencies were used to obtain parameter estimates through functions adapted from a previous study^59^ using the R program (version 4.0.0)^60^. This information is presented in Supplementary Table S4 online. The prevalence $\widehat{\pi }$ of each behaviour in the DQ group was given by:

$$\widehat{\pi }=\frac{Number\, of\, \mathrm{Yes }\,responses }{Total\, number\, of\, responses}.$$

The prevalence estimate of each behaviour in each ECWM group was given by:

$$\widehat{\pi }\mathrm{ECWM }=\frac{ ( \widehat{\lambda }+ p-1) }{(2p-1)}$$

where $\widehat{\lambda }$ is the observed proportion of option A responses and *p* is the randomisation probability^37^. We estimated the values for *p* for ECWM1 (*p1*) as 0.256, and for ECWM2 (*p2* = *1 - p1)* as 0.744, from data available on livebirths by month in England for 1938–1991^61^. Likelihood ratio tests G^2^ (the G-test of goodness of fit) were conducted to compare estimates across the two ECWM groups to detect if there was a systematic bias for one of the presented options^37^. If the difference between the two groups was statistically significant (p < 0.05), then the prevalence estimates were not pooled, and no further analysis was conducted as the estimates were considered as biased and unreliable. If respondents understood and adhered to CWM instructions, the group-specific prevalence estimates πECWM1 and πECWM2 would only differ by chance. That is, the difference would not be statistically significant (p ≥ 0.05), and the estimates can be pooled and used in subsequent analysis.

To compare and verify if the difference between prevalence estimates for sensitive behaviours obtained via DQ and ECWM were statistically significant, a two-proportion Z test was conducted. Since the sample size at hand was sufficiently large, Fisher’s exact binomial test was replaced with the normal approximation to the binomial distribution.

To examine the relationships between the behaviours and the predictor variables, “modified” logistic regression analysis was conducted including DQ data using the RRreg package in R (version 0.7.4)^62^. For this step, the ECWM 1 and ECWM 2 groups were combined into a single ECWM group, provided reliable estimates were obtained. This involved flipping the response codes of one of the ECWM groups, which is possible as the randomisation probabilities of the two groups are exactly reversed. Coding of ECWM2 was flipped within this study. The randomisation probability of the un-flipped group (ECWM1) was used in all the logistic regression analysis. The prevalence estimates were regressed on the predictor variables while controlling for effect-coded questioning format (− 1 = DQ; + 1 = ECWM) and a RRgroup variable that determines which question format was used on the side of the dependent variable (0 = DQ, 1 = ECWM; this coding is also internally fixed by the RRreg package).

### Supplementary Information

Supplementary Information.

## Data Availability

The datasets used and/or analyzed during the current study are available from the corresponding author on reasonable request. Study materials and outputs are available at https://osf.io/2kcuw/.

## Code availability

All coding scripts used for data analysis are available at https://osf.io/2kcuw/.
