# Supplementary material for: Falsification of home rapid antigen lateral flow tests during the COVID-19 pandemic
Source: Sci Rep. 2024 Feb 9;14:3322. doi: 10.1038/s41598-024-53383-8 (PMC10858045; doi:10.1038/s41598-024-53383-8)
Supplement: Supplementary file 1 — Supplementary Information. [file 41598_2024_53383_MOESM1_ESM.docx]

**Supplementary Information File**

**Title of the manuscript:** Falsification of home rapid antigen lateral flow tests during the COVID-19 pandemic.

**List of authors and affiliations**

Devashish Ray^a*^ (DR)

Raenhha Dhami^a^ (RD)

Jan Lecouturier^a^ (JL)

Laura J McGowan^a^ (LM)

Aritra Mukherjee^e^ (AM)

Ivo Vlaev^b^ (IV)

Michael P. Kelly ^a, c^  (MK)

Falko F. Sniehotta ^a, d^ (FS)

**^a^**NIHR Policy Research Unit in Behavioural Science - Population Health Sciences Institute, Faculty of Medical Sciences, Newcastle University, Newcastle upon Tyne, UK

**^b^**NIHR Policy Research Unit in Behavioural Science – Behavioural Science Group, Warwick Business School, University of Warwick, Coventry, UK

**^c^**Primary Care Unit, Department of Public Health and Primary Care, University of Cambridge, Forvie Site, Cambridge CB2 0SR. UK

**^d^**Department of Public Health, Preventive and Social Medicine, Center for Preventive Medicine and Digital Health Baden-Wuerttemberg, Heidelberg University, Germany

**^e^**Biostatistics Research Group, Population Health Sciences Institute, Newcastle University, Newcastle upon Tyne, UK

**Table S1.** Demographic characteristics of survey participants (N = 1577). Only the variables listed in the parenthesis (age, and sex/ gender) were the demographic variables used in the final analysis.

| **Category** | Percentages (in parentheses: average for England and Wales, 2021) | | **Category** |  | Percentages |  |
| --- | --- | --- | --- | --- | --- | --- |
| **Sex/ gender** | *Male* | 42.74 (49) | **Social grade** | *ABC1* | 65.31 |  |
|  | *Female* | 57.26 (51) |  | *C2DE* | 34.69 |  |
| **Age (in Years)** | *18-24* | 11.10 (11) | **Key worker** | *Yes* | 35.13 |  |
|  | *25-34* | 21.24 (17) |  | *No* | 37.79 |  |
|  | *35-44* | 18.83 (16) |  | *N/A* | 27.08 |  |
|  | *45-54* | 16.74 (17) | **Children in household** | *0* | 73.87 |  |
|  | *55-64* | 16.11 (15) |  | *1+* | 24.41 |  |
|  | *65+* | 15.98 (23) | **Chronic illness (self)** | *Yes* | 21.75 |  |
| **Education level** | *Low* | 17.25 |  | *No* | 72.99 |  |
|  | *Medium* | 37.16 | **Chronic illness (household excl. self)** | *Yes* | 21.88 |  |
|  | *High (University degree)* | 45.47 |  | *No* | 75.21 |  |
| **Ethnicity** | *White* | 85.86 (81.7%) | **Household status** | *Live alone* | 79.96 |  |
|  | *Ethnic minority* | 11.16 |  | *Live with others* | 16.42 |  |
| **Region in England** | *Northeast* | 5.33 | **Gender identity** | *Cis* | 96.32 |  |
|  | *Northwest* | 12.62 |  | *Trans* | 0.70 |  |
|  | *Yorkshire and Humber* | 9.51 |  | | |  |
|  | *East Midlands* | 10.02 |  |  |  |  |
|  | *West Midlands* | 8.50 |  |  |  |  |
|  | *East of England* | 11.35 |  |  |  |  |
|  | *London* | 11.10 |  |  |  |  |
|  | *Southeast* | 19.66 |  |  |  |  |
|  | *Southwest* | 11.92 |  |  |  |  |

**Figure S1.** Bar graph displaying participant perceptions regarding what may motivate people to report a positive LFT result as negative.

**Figure S2.** Bar graph displaying participant perceptions regarding what may motivate people to report a negative LFT result as positive.

**Table S2**. Modified Logistic Regression using RRreg package across the three valid sensitive behaviours (falsification behaviours) with demographic variables - while controlling for questioning format (added as an effect-coded variable). This modified regression approach accounts for the discrepancy between the observed responses and the true latent states in the E(CWM).

| **Demographic variables** | ***Odds ratio*** | | ***95% CI*** | | | | ***p-value*** | |  |
| --- | --- | --- | --- | --- | --- | --- | --- | --- | --- |
|  |  | | **Lower** | | **Upper** | |  | |  |
| ***FB*1: Reported a negative result without doing a test** | | | | | | | | |  |
| Gender | | 1.457 | | 0.79 | | 2.689 | | 0.225 | |
| Age | | 1.312 | | 0.679 | | 2.534 | | 0.423 | |
| Social Grade | | 1.444 | | 0.743 | | 2.809 | | 0.269 | |
| Key worker status | | 0.607 | | 0.30 | | 1.229 | | 0.153 | |
| Education | | 0.931 | | 0.608 | | 1.427 | | 0.743 | |
| ***FB*2: Reported a positive test result as negative** | | | | | | | | |  |
| Gender | | 0.595 | | 0.259 | | 1.369 | | 0.204 | |
| Age | | 0.836 | | 0.372 | | 1.876 | | 0.664 | |
| Social Grade | | 1.502 | | 0.631 | | 3.573 | | 0.347 | |
| Key worker status | | 0.835 | | 0.329 | | 2.116 | | 0.699 | |
| Education | | 0.968 | | 0.581 | | 1.613 | | 0.899 | |
| ***FB*3: Reported a positive result after having produced a fake positive test** | | | | | | | | |  |
| Gender | | 0.287 | | 0.055 | | 1.501 | | 0.194 | |
| Age | | **0.176** | | **0.034** | | **0.903** | | **0.023*** | |
| Social Grade | | 4.753 | | 0.284 | | 0.868 | | 0.072 | |
| Key worker status | | 0.196 | | 0.022 | | 1.721 | | 0.085 | |
| Education | | 0.659 | | 0.261 | | 1.662 | | 0.373 | |

* p < 0.05; ** p < 0.01; *** p < 0.001

**Note**: Analysis for the variable of key worker status was conducted on 1027 subjects – after the exclusion of NA coded as ‘3’ by the market research company. The remaining variables had 1405 participants.

**Table S3.** “Modified” Logistic Regression using RRreg package to test if questioning format moderates’ relationship between predictors and sensitive behaviours. Questioning format is introduced as an effect-coded interaction term. This “modified” regression approach accounts for the discrepancy between the observed responses and the true latent states in the E(CWM).

|  | **ECWM** | | | | | | **DQ** | | | **Test of difference** | |
| --- | --- | --- | --- | --- | --- | --- | --- | --- | --- | --- | --- |
|  | ***Odds Ratio*** | | | ***95% CI***  ***Lower         Upper*** | | | ***Odds ratio*** | ***95% CI***  ***Lower       Upper*** | | ***p -value*** | |
| **FB1: Reported a negative test result without doing a test** | | | | | | | | | | | |
| Perception of risk (self) | | 1.015 | | | 0.536 | 1.919 | 1.220 | 0.82 | 1.787 | 0.641 |  |
| Perception of risk (others) | | 1.001 | | | 0.603 | 1.661 | 1.235 | 0.828 | 1.832 | 0.526 |  |
| Confidence in govt. | | 1.115 | | | 0.678 | 1.835 | 0.796 | 0.562 | 1.163 | 0.31 |  |
| Belief in test | | 0.814 | | | 0.324 | 2.049 | 1.043 | 0.697 | 1.676 | 0.584 |  |
| Perceived ease | | 1.308 | | | 0.798 | 2.142 | 1.063 | 0.701 | 1.649 | 0.534 |  |
| Subjective norm | | **1.141** | | | **0.745** | **1.747** | **0.600** | **0.432** | **0.847** | **0.042*** |  |
| Anticipated regret | | 1.214 | | | 0.762 | 1.933 | 0.658 | 0.463 | 0.978 | 0.08 |  |
| Moral norm | | **1.471** | | | **0.976** | **2.216** | **0.585** | **0.426** | **0.820** | **0.004**** |  |
| **FB2: Reported a positive test result as negative** | | | | | | | | | | | |
| Perception of risk (self) | | 2.283 | | | 0.496 | 10.520 | 1.380 | 0.881 | 2.127 | 0.481 |  |
| Perception of risk (others) | | 1.078 | | | 0.528 | 2.201 | 1.007 | 0.626 | 1.591 | 0.874 |  |
| Confidence in govt. | | 1.351 | | | 0.753 | 2.423 | 0.778 | 0.525 | 1.203 | 0.165 |  |
| Belief in test | | 1.444 | | | 0.903 | 2.310 | 1.117 | 0.694 | 1.989 | 0.484 |  |
| Perceived ease | | 1.705 | | | 0.931 | 3.121 | 0.870 | 0.547 | 1.417 | 0.089 |  |
| Subjective norm | | **1.704** | | | **1.066** | **2.723** | **0.548** | **0.379** | **0.804** | **0.002 **** |  |
| Anticipated regret | | **1.769** | | | **1.074** | **2.913** | **0.574** | **0.394** | **0.872** | **0.007**** |  |
| Moral norm | | 1.363 | | | 0.779 | 2.383 | 0.524 | 0.370 | 0.755 | 0.062 |  |
| **FB3: Reported a positive test result after having produced a fake positive test** | | | | | | | | | | | |
| Perception of risk (self) | | | 0.463 | | 0.089 | 2.405 | 2.213 | 1.084 | 4.667 | 0.546 |  |
| Perception of risk (others) | | | 3.894 | | 0.510 | 29.70 | 1.351 | 0.624 | 2.902 | 0.337 |  |
| Confidence in govt. | | | 19.620 | | 0.002 | 230280 | 0.507 | 0.278 | 0.953 | 0.152 |  |
| Belief in test | | | 0.718 | | 0.030 | 17.387 | 1.671 | 0.675 | 5.677 | 0.488 |  |
| Perceived ease | | | 0.671 | | 0.173 | 2.601 | 0.518 | 0.254 | 1.097 | 0.753 |  |
| Subjective norm | | | 0.268 | | 0.003 | 22.702 | 0.358 | 0.194 | 0.639 | 0.883 |  |
| Anticipated regret | | | 2.163 | | 0.925 | 5.055 | 0.379 | 0.219 | 0.663 | 0.096 |  |
| Moral norm | | | 3.026 | | 0.321 | 28.506 | 0.379 | 0.220 | 0.646 | 0.164 |  |

* p < 0.05; ** p < 0.01; *** p < 0.001

This analysis sought to determine if questioning format (direct or indirect technique) moderated the relationship between the predictors and types of falsification behaviours. We observed that this was indeed true for subjective and moral norm for FB1 as well as subjective norm and anticipated regret for FB2. However, only subjective norm for FB1 was statistically significant in the combined (DQ+ECWM) analysis (as shown in Table 4 in main manuscript). This would suggest that the relationship between subjective norm and FB1 was indeed moderated by the questioning format. Specifically, this association appears to be affected by socially desirable responding in the DQ format.

**Table S4.** Respondents’ observed answer frequencies to the sensitive questions [DQ=direct questioning; ECWM= extended crosswise model]. ‘Yes’ and ‘No’ were response options for those in the DQ conditions. Option A – ‘My answer is Yes/No to both questions,’ and Option B – ‘My Answer is Yes to one & No to other, irrespective of which one’ were response option for those in the ECWM 1 and 2 conditions. These frequencies were used to calculate respective question format dependant prevalence estimates.

| **Question** | **Questioning**  **method** | **Yes/Option A** | **No/Option B** | **Total (n)** |
| --- | --- | --- | --- | --- |
| ***FB1:*** *Reported a negative test without conducting a test* | DQ | 30 | 493 | 523 |
|  | ECWM 1 | 322 | 182 | 504 |
|  | ECWM 2 | 169 | 340 | 509 |
| ***FB2:*** *Reported a positive test result as negative* | DQ | 24 | 499 | 523 |
|  | ECWM 1 | 345 | 147 | 492 |
|  | ECWM 2 | 161 | 340 | 501 |
| ***FB3:*** *Reported a positive test result after having produced a fake positive test* | DQ | 9 | 516 | 525 |
|  | ECWM 1 | 352 | 141 | 493 |
|  | ECWM 2 | 146 | 357 | 503 |
| ***FB4:*** *Shared information about the test for someone else to report it as their own test* | DQ | 11 | 513 | 524 |
|  | ECWM 1 | 289 | 203 | 493 |
|  | ECWM 2 | 138 | 363 | 502 |

**Methods. Implementation of the Extended Crosswise model**

**Instructions to respondents.**

In this section, we use a special method to ask questions about COVID-19 home lateral flow testing and reporting of test results**.** Please read the instructions carefully.

You will see two questions that are presented together in a block. For example,

1. Is your mother’s birth month May, June, or July? (If you don't know/ are not sure, please use the birthday of another person you know).

2. Have you ever reported a negative test result without actually doing the test?

Instead of answering each question separately, you only have to indicate whether:

Option A: Your answer is Yes to both questions or your answer is No to both questions

***OR***

Option B: Your answer is Yes to one question and No to the other question.

Your anonymity remains protected because we do not know your answers to the individual questions. We also do not know, for example, your mother’s birth month. Therefore, we cannot know which of the two statements is either true or false for you, and thus, your anonymity is assured. For each block, please consider the two questions together and select **only one** option response.

**Sensitive question 1:**

(i) For ECWM group 1: Is your mother’s birth month May, June, or July?

(If you don't know/ are not sure, please use the birthday of another person you know)

(For ECWM group 2 it was: Is your mother’s birth month August, September, October, November, December, January, February, March, or April?)

(ii) Have you ever reported a negative home lateral flow test result without actually doing the test?

Option A  My answer is Yes to both questions *or* No to both questions.

Option B  My answer is Yes to one question *and* No to the other question (it does not matter which one)

**Sensitive question 2:**

(i) For ECWM group 1: Is your father’s birth month May, June, or July?

(If you don't know/ are not sure, please use the birthday of another person you know)

(For ECWM group 2 it was: Is your father’s birth month August, September, October, November, December, January, February, March, or April?)

(ii) Have you ever reported a negative home lateral flow test result when it was positive?

(The answer options were presented in exactly the same way for all four sensitive questions).

**Sensitive question 3:**

(i) For ECWM group 1: Is ***your*** birthday in May, June, or July?

(For ECWM group 2 it was: Is ***your*** birthday in August, September, October, November, December, January, February, March, or April?

(ii) Have you ever reported a positive home lateral flow test result when it was negative, or reported a positive result after having produced a fake positive result (for example, by using liquids such as a soft drink or other drinks)?

**Sensitive question 4:**

(i) For ECWM group 1: Think of a ***brother, sister, friend, or a person*** whose birth month you know. Is his/ her birthday in May, June, or July?

(For ECWM group 2 it was: Think of a ***brother, sister, friend, or a person*** whose birth month you know. Is his/ her birthday in August, September, October, November, December, January, February, March, or April?)

(ii) Have you ever shared information from your lateral flow test (e.g., the test strip ID or a picture of the test strip) for someone else to report it as their test?

**References**

Kaufmann, T. H., Lilleholt, L., Böhm, R., Zettler, I., & Heck, D. W. (2022). Sensitive attitudes and adherence to recommendations during the COVID-19 pandemic: Comparing direct and indirect questioning techniques. *Personality and Individual Differences*, *190*, 111525. https://doi.org/10.1016/j.paid.2022.111525
